# Histone demethylase JMJD2D emerges as a novel prognostic biomarker and exhibits correlation with immune infiltration in lung adenocarcinoma

**DOI:** 10.1007/s12672-025-02871-y

**Published:** 2025-06-12

**Authors:** Bona Liu, Hui Hao, Zhen Wang, Yingchun Li, Cheng Du, Jian Ming, Shuang Zhang, Lin Zhou, Dazhi Liu

**Affiliations:** 1 Department of Oncology, General Hospital of Northern Theater Command, Shenyang, China; 2 Department of Medical Oncology, Hebei Cangzhou People’s Hospital, Cangzhou, China; 3 Department of Oncology, General Hospital of Northern Theater Command, Dalian Medical University, Shenyang, China; 4 Department of Pathology, General Hospital of Northern Theater Command, Shenyang, China; 5 Department of Military Patient Management, General Hospital of Northern Theater Command, Shenyang, China; 6 Department of Thoracic Surgery, Yuebei People’s Hospital Affiliated to Shantou University Medical College, Shaoguan, China; 7 Department of Thoracic Surgery, General Hospital of Northern Theater Command, Shenyang, China

**Keywords:** JMJD2D, Lung adenocarcinoma (LUAD), Overall survival, Immune cell infiltration, Tumor mutation burden (TMB)

## Abstract

**Background:**

Lysine demethylase 4D (KDM4D or JMJD2D) plays a significant role in tumorigenesis, development, and poor clinical outcomes. However, its roles in lung adenocarcinoma (LUAD) remains unclear. This study aimed to identify the role and molecular mechanisms of JMJD2D in LUAD.

**Methods:**

The study investigated the correlation of JMJD2D with tumor development, immune cell infiltration, response to antitumor therapy, tumor mutation burden and prognostic values.

**Results:**

JMJD2D had high expression in LUAD. High-JMJD2D expression was associated with poor survival outcomes and the T stage of LUAD patients. Furthermore, high-JMJD2D expression was linked to the decreased immune-related processes, associated with the increased Tregs and CD40 expression. Additionally, high-JMJD2D expression was associated with frequent alterations with higher TMB and resistance to BMS.708,163, Roscovitine, and Pyrimethamine, but sensitivities to ATRA, Bosutinib, and JNK. Inhibitor. VIII. Moreover, the study identified eight JMJD2D-related genes as a prognostic signature and constructed a predictive nomogram based on independent prognostic factors.

**Conclusion:**

JMJD2D acts as an oncogene in LUAD and is involved in tumorigenesis, development, and poor clinical outcomes. Therefore, JMJD2D may serve as a potential prognostic biomarker in diagnosis and treatment of LUAD. The study emphasizes the importance of the molecular mechanisms of JMJD2D in LUAD.

**Supplementary Information:**

The online version contains supplementary material available at 10.1007/s12672-025-02871-y.

## Introduction

Lung cancer is a serious malignancy that is estimated to cause approximately 2.2 million new cases and 1.8 million deaths in 2020 [1]. Lung cancer is the leading cause of cancer-related morbidity and mortality in China in the past decades, resulting in a significant health, financial, and societal burden [2, 3]. The overall five-year survival rates of patients with lung cancer are only 75.2%, 45.25%, 24%, and 8%, respectively, for stages I to IV [2]. According to histological classification, lung cancer is divided into small-cell lung carcinoma (SCLC) and non-small-cell lung carcinoma (NSCLC), Small-cell lung carcinoma (SCLC) and non-small-cell lung carcinoma (NSCLC) [4]. Approximately 85% of cases are NSCLC, which is further classified into lung squamous cell carcinoma (LUSC), lung adenocarcinoma (LUAD), and large-cell carcinoma (LCC) [5]. Although the introduction novel agents and the application of predictive biomarkers for patients with advanced NSCLC, the long-term survival outcomes remain poor [6–8]. Given the poor clinical outcomes of patients with NSCLC, it is crucial to identify additional potential biomarkers for the diagnosis and treatment of patients with NSCLC.

Epigenetic modifications, including DNA or RNA methylation, non-coding RNAs, chromatin remodeling, and histone modifications, are involved in tumorigenesis, progression, metastasis, and drug resistance, all of which are closely associated with tumor heterogeneity in lung cancer [9–11]. Of these epigenetic modifications, histone modifications play important roles in the regulation of chromatin state, gene expression, and other nuclear events in the various cancer types [12]. Histone modifications represent a crucial epigenetic mechanism, encompassing acetylation, phosphorylation, methylation, ADP ribosylation, ubiquitination, and citrullination, and more [13–16]. Numerous studies have indicated that histone methylation and demethylation are involved in tumor progression and drug resistance [17–19]. Histone demethylases contain two types of demethylases, including histone lysine demethylases and histone arginine demethylases, which consist of the lysine-specific demethylase (LSD) family, the Jumonji C (JmjC)-domain-containing demethylase (JMJD) family and the histone arginine demethylases [20]. JMJD2D, also named KDM4D, is a demethylase that targets histone H3 on lysines 9 and 36 and histone H1.4 on lysine 26 [21]. JMJD2D is widely overexpressed in various tumors. For example, upregulated JMJD2D is observed in human liver cancers and is associated with poor survival [22]. Moreover, it also reported that upregulated JMJD2D is found in colorectal cancer (CRC) and promotes tumor progression [23–25]. JMJD2D is also overexpressed in prostate tumors and promotes prostate tumorigenesis [26]. Upregulation of JMDJ2D in gastrointestinal stromal tumors (GST) is observed and promotes tumor progression [27]. JMJD2D is also overexpressed in acute myeloid leukemia (AML) and is linked to poor overall survival in AML patients [28]. A study has reported that JMJD2D expression is associated with the metastatic spread of lung carcinomas [29]. However, studies on JMJD2D in lung cancer, particularly LUAD, remain limited. Given the scarcity of previous research, we conducted a comprehensive bioinformatics analysis to investigate the expression and molecular characteristics of JMJD2D in LUAD, aiming to identify potential diagnostic and therapeutic targets for the disease.

In the present study, we explored the correlation of JMJD2D with tumor development, immune cell infiltration, drug resistance, and prognosis values based on the data of LUAD from The Cancer Genome Atlas (TCGA) and Gene Expression Omnibus (GEO). Furthermore, we also identified the JMJD2D-related risk signature and then developed a predictive nomogram for patients with LUAD.

## Methods

### Human tissue microarray analysis of JMJD2D

Human LUAD tumor tissues microarray (AF-LucSur2202) containing 80 patients with LUAD and adjacent normal tissues were obtained from Yunnan Yantai Biotechnology Co. Ltd. Each sample plot with a diameter of 1.5 mm and a thinness of 4-µm was prepared based on a standard method. Immunohistochemistry (IHC) staining was performed using the anti-JMJD2D antibody (1:300, 22591-1-AP, Proteintech, Wuhan). The IHC score of each sample, which ranged from 0 to 12, was calculated according to the intensity of the nucleic staining (no staining = 0; weak brown staining = 1, moderate brown staining = 2 and strong brown staining = 3) and the extent of stained cells (0–5%=0, 5–25%=1, 26–50%=2, 51–75%=3 and 76–100%=4) as previously described [30].

### Data sources and processing

Transcriptome profiles, along with corresponding clinical information and somatic mutation data of LUAD patients, were acquired from the Cancer Genome Atlas (TCGA, https://portal.gdc.cancer.gov/). The transcriptome profiles of 535 LUAD tumors and 59 normal samples were retrieved using the University of California, Santa Crus (UCSC Xena, https://xena.ucsc.edu/), while the corresponding clinical information was obtained from the TCGA-LUAD project within the TCGA database through the cBioPortal platform at (https://www.cbioportal.org/). After quality assessments, data integration, batch correction, and normalization, a total of 479 LUAD tumors and 59 normal samples were included for subsequent analyses. Additionally, the GSE68465 dataset, which comprises the transcriptome profiles and corresponding clinical information of 462 LUAD patients, was obtained from the Gene Expression Omnibus (GEO, https://www.ncbi.nlm.nih.gov/geo/) and generated by the GPL96 platform. Following quality control and normalization, 442 LUAD specimens with complete clinical information were incorporated into this study.

### Differential expression of JMJD2D across multiple cancer types

The transcriptome profiles of sixteen tumors were downloaded from the TCGA database, including bladder cancer (BLCA), breast cancer (BRCA), cervical cancer (CESC), colon cancer (COAD), esophageal cancer (ESCA), head and neck cancer (HNSC), kidney clear cell carcinoma (KIRC), liver cancer (LIHC), lung adenocarcinoma (LUAD), lung squamous cell carcinoma (LUSC), pancreatic cancer (PAAD), prostate cancer (PRAD), rectal cancer (READ), stomach cancer (STAD), thyroid cancer (THCA), and glioblastoma (GBM). The Wilcoxon rank-sum test was used to compare JMJD2D expression between tumor and normal samples across all cancer types, and the visualization of results was performed using the “ggpubr” package (version 0.4.0) in R.

### Survival analysis

The transcriptome profiles of LUAD patients, along with corresponding clinical information such as survival time and survival status, were integrated using R. The optimal cutoff value of JMJD2D expression was determined using the “survminer” (version 0.4.9) package in R based on the maximally selected rank statistics from the “maxstat” R package, which is an outcome-oriented method, provides a cutoff value that corresponds to the most significant relationship with survival [31]. Then, patients were stratified into high-JMJD2D and low-JMJD2D groups according to the optimal cutoff value (Fig. 3C). Kaplan-Meier (KM) survival analysis and log-rank test were performed using the “survival” (version 3.2-7) package to compare overall survival (OS) differences between the high-JMJD2D and low-JMJD2D groups (Fig. 3D). Additionally, the Kruskal-Wallis test was employed to assess differences in age, gender, survival status, pathological stage, American Joint Committee on Cancer (AJCC) TNM stage, and other clinical parameters between the high-JMJD2D and low-JMJD2D groups (Fig. 3E). This comprehensive analysis aims to elucidate the potential prognostic significance of JMJD2D expression in LUAD patients.

### Identification of the differentially expressed genes (DEGs)

“Limma” R package (version 3.46.0) [32] was utilized to identify Differentially Expressed Genes (DEGs) between tumor and normal specimens, as well as between high and low JMJD2D expression groups. DEGs were screened based on cutoff values of absolute log-fold change (FC) > 0.5 and adjusted *p*-value < 0.05 (Table S5). “ggplot2” R package (version 3.3.2) (Fig. 8A) [33] and “Pheatmap” R package (version 1.0.12) [34] were employed for visualization purposes (Fig. 8B). The DEGs, as well as the top 50 DEGs ranked by adjusted p-value, were visualized using these packages, providing insights into gene expression alterations associated with tumor status and JMJD2D expression levels. This approach enhances our understanding of the molecular mechanisms underlying LUAD progression and JMJD2D-associated pathways.

### Gene set enrichment analysis (GSEA)

GSEA was employed to explore the immune-related pathways associated with JMJD2D expression. The “org.Hs.eg.db” R package (version 3.12.0) [35] was used to retrieve the Entrez-ID of each Differentially Expressed Gene (DEG). Subsequently, the “clusterProfiler” R package (version 3.18.1) [36] was employed to conduct the Gene Ontology (GO) analysis. The top 10 significantly enriched pathways, ranked by adjusted P-value were visualized using the “enrichplot” R package (version 1.10.2) [37]. This visualization enhances the interpretation of the GO analysis results, providing insight into the immune-related pathways that are potentially regulated by JMJD2D expression. This comprehensive approach contributes to our understanding of the immunological implications of JMJD2D in the context of LUAD.

### Immune cell infiltration analysis

The “estimate” R package (version 1.0.13) was employed to calculate the immune score, stromal score, and estimate score for each sample, providing valuable insights into the tumor microenvironment. Besides, the “immunedeconv” R package (version 2.0.4) [38] employed the quantiseq algorithm to evaluate the distribution of infiltrated immune cells based on the transcriptome profiles. Moreover, CIBERSORT (version 1.03), a deconvolution algorithm with a leukocyte signature matrix (LM22) consisting of 547 genes distinguishing 22 immune populations [39], was utilized to assess the distribution of 22 types of infiltrating immune cells based on transcriptome profiles. Differences in cell fractions between the two groups were determined via the Wilcoxon rank-sum test and visualized using the “vioplot” R package (version 0.3.7). Moreover, differences in cell fractions were assessed using the Wilcoxon rank-sum test and visualized using the “ggpubr” R package (version 0.4.0) [40]. A comprehensive analysis was performed on 43 immune checkpoint-related genes (ICRGs) obtained from a previous study [41], wherein differences in the expression of 25 ICRGs were evaluated using the Wilcoxon rank-sum test. These analyses shed light on the immune landscape and potential immunotherapeutic targets in LUAD.

### Prediction of the therapeutic responses to immunotherapy

The Tumor Immune Dysfunction and Exclusion (TIDE) algorithm is a computational method designed to model two primary mechanisms of tumor immune evasion: high infiltration of cytotoxic T lymphocytes (CTL) inducing T cell dysfunction within tumors, and low infiltration of CTL resulting in reduced T cell infiltration [42]. To evaluate the TIDE score for each sample, data from the TIDE database (http://tide.dfci.harvard.edu/) were utilized. Subsequently, differences in TIDE scores between different groups were determined using the Wilcoxon rank-sum test.

### Tumor mutation burden (TMB) analysis

The Mutation Annotation Format (MAF) profiles of simple nucleotide variation were downloaded from the TCGA database (https://portal.gdc.cancer.gov/cart). The Tumor Mutational Burden (TMB) score for each sample was assessed and analyzed using the “maftools” R package (version 2.6.05) [43]. Subsequently, differences in TMB between the high-JMJD2D and low-JMJD2D groups were determined via the Wilcoxon rank-sum test and visualized using the “ggpubr” R package (version 0.4.0). The relationship between TMB and JMJD2D expression was determined by Spearman correlation analysis and visualized using the “ggExtra” R package (version 0.10.0). Additionally, the top 20 frequent mutation genes between high-JMJD2D and low-JMJD2D groups were visualized using the “oncoplot” function, providing insights into the mutational landscape associated with JMJD2D expression levels.

### Drug sensitivity analysis

The information on 138 common targeted drugs was obtained from the Genomics of Drug Sensitivity in Cancer (GDSC, https://www.cancerrxgene.org/) database. Then, the “pRRophetic” R package (version 0.5) [44] was performed to calculate the Half-maximal inhibitory concentration (IC50) value of each sample based on the gene expression profiles. The lower IC50 value indicates greater sensitivity to the targeted drug. The correlation between JMJD2D expression and the IC50 value of drugs was visualized using the “ggplot2” R package. Furthermore, differences in IC50 values between the high-JMJD2D and low-JMJD2D expression groups were detected by the Wilcoxon rank-sum test. This comprehensive analysis provides insights into the potential associations between JMJD2D expression levels and drug sensitivity, aiding in the identification of personalized therapeutic strategies for LUAD patients.

### Identification and validation of the JMJD2D-related prognosis signature in LUAD

Differentially expressed JMJD2D-related genes (DE-JMJD2D-RGs) were obtained using the “venn” R package (version 1.11) by intersecting the DEGs identified from tumor compared with normal samples and from the high-JMJD2D compared with low-JMJD2D groups, respectively (Fig. 8C). Subsequently, univariate Cox analysis was performed using the “survival” R package (version 3.2-7) to identify the prognostic DE-JMJD2D-RGs, and the results were visualized using the “forestplot” R package (version 2.0.1). The DE-JMJD2D-RGs with p-value < 0.05 were then incorporated into a least absolute shrinkage and selection operator (LASSO) regression model to prevent overfitting using the “glmnet” package in R (version 4.1-1). A prognostic signature comprising eight DE-JMJD2D-RGs was constructed through LASSO regression analysis. The JMJD2D-related risk score was calculated according to the formula, risk score = $\:\sum\:_{i=1}^{n}coef\:\left(genei\right)*expr\:\left(genei\right)$. Here, coef represented the coefficient of each gene, and expr represented the expression level of each gene. Then, all patients of the TCGA-LUAD cohort were divided into high-risk and low-risk groups based on the median value of the risk score (Fig. 8H). The overall survival (OS) between high-risk and low-risk groups was conducted using the “survminer” package in R (version 0.4.8) (Fig. 8I). Furthermore, Kaplan-Meier (KM) analysis based on the risk score and receiver operating characteristic (ROC) curve was drawn using the “survivalROC” R package (version 1.0.3) to evaluate the performance of the risk model (Fig. 8J). Additionally, the correlation between risk score and clinical information, such as age, gender, survival status, pathological stage, and AJCC TNM staging, was determined using the Wilcoxon rank-sum test or the Kruskal-Wallis test ((Fig. 8G). Moreover, the GSE68465 dataset served as an external validation set to validate the robustness of the risk model (Fig. 8K-M). This comprehensive analysis aims to develop and validate a prognostic model based on JMJD2D-related risk scores, providing valuable insights into LUAD patient prognosis and potential clinical applications.

### Construction of a nomogram

The JMJD2D-related risk score and clinical information were incorporated into univariate and multivariate Cox analyses to identify independent prognostic factors for LUAD. Factors with a *p*-value < 0.05 were selected as independent factors. Subsequently, these factors were utilized to construct a nomogram using the “rms” package in R (version 6.2.0). The discrimination ability of the nomogram was evaluated using the Harrell concordance index (C-index), which assesses the model’s ability to distinguish between patients with different survival outcomes. Additionally, calibration curves were generated to assess the agreement between the predicted and observed probabilities of survival. This integrated approach aims to develop a reliable prognostic tool for LUAD patients, facilitating personalized treatment decisions and improving clinical outcomes.

## Results

### Identification of JMJD2D expression across various Cancer types

**Fig. 1:**
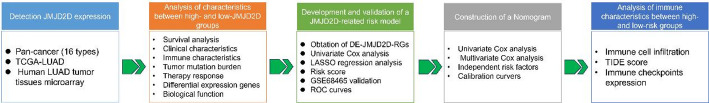
The flowchart of outlining the methodology and key steps undertaken in this study

**Fig. 2:**
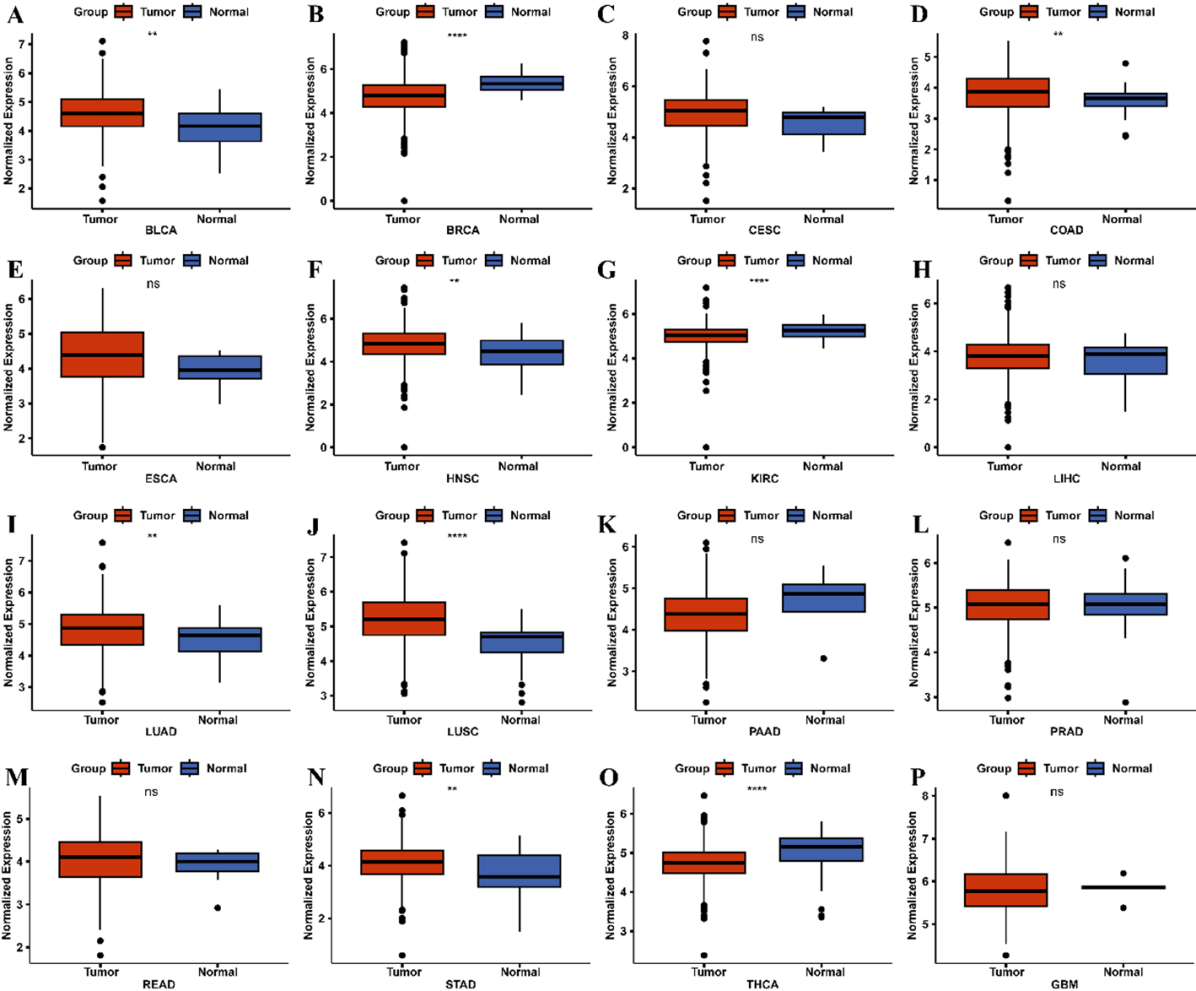
Identification of JMJD2D expression across various cancer types. A-P. The expression levels of JMJD2D were analyzed across multiple cancer types, including BLCA, COAD, HNSC, LUAD, LUSC, STAD, BRCA, KIRC, and THCA. * *P* < 0.05, ** *P* < 0.01, *** *P* < 0.001, and **** *P* < 0.0001

The study design is illustrated in Fig. 1. Differential expression analysis of JMJD2D across sixteen tumor types, as depicted in Fig. 2, revealed that JMJD2D expression was significantly elevated in certain cancers compared to normal tissues. Specifically, high JMJD2D expression was observed in BLCA, COAD, HNSC, LUAD, LUSC, and STAD. Conversely, whereas low JMJD2D expression was noted in BRCA, KIRC, and THCA. These findings suggest that JMJD2D may exhibit dual roles in tumorigenesis, with its expression varying across different cancer types. Notably, JMJD2D appears to function as an oncogene in lung cancer, particularly in LUAD and LUSC.

### High expression of JMJD2D associated with poor overall survival of LUAD patients

**Fig. 3:**
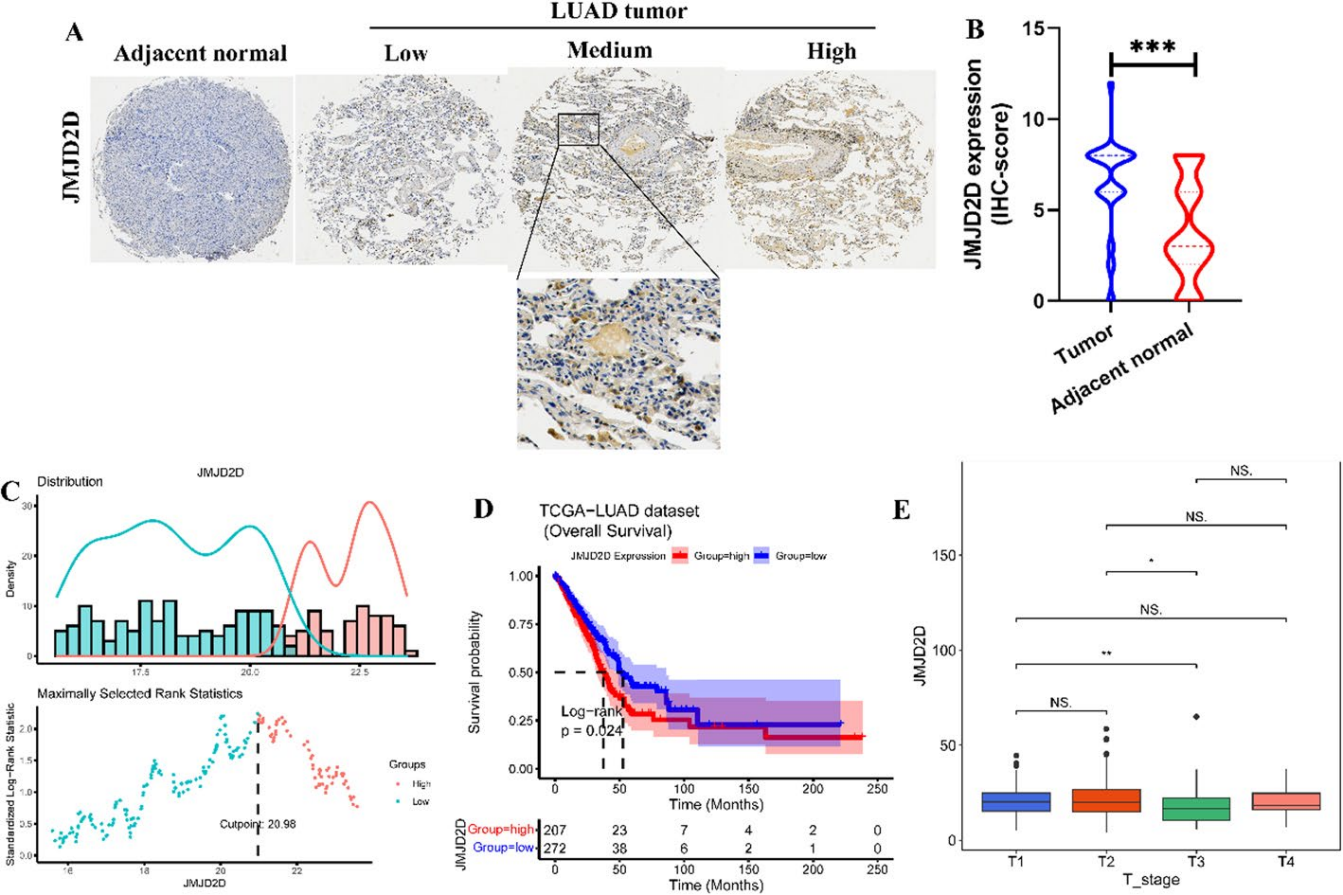
High expression of JMJD2D associated with poor overall survival of LUAD patients. A. IHC staining was performed using an anti-JMJD2D antibody on human LUAD tumor samples and adjacent normal samples at 60x and 100x magnification. B. Statistical analysis of the immunohistochemical staining results. *** *P* < 0.001. C. Determination of the optimal cutoff value of 20.98 based on maximally selected rank statistics. D. Kaplan-Meier survival curves depicting the overall survival of patients with high- and low-JMJD2D expression according to the optimal cutoff value. E. Analysis of the correlation between JMJD2D expression and the AJCC-T stage. * *P* < 0.05, and ** *P* < 0.01

To investigate the role of JMJD2D in LUAD, IHC staining was performed to detect the expression of JMJD2D in LUAD specimens. As shown in Fig. 3A-B, JMJD2D exhibited higher expression levels in LUAD tumor samples compared to adjacent normal tissues. Given this observation, we focused on the role of JMJD2D in the LUAD in the subsequent analyses. For our study, a total of 479 LUAD patients with complete clinical information were included, source from the TCGA database. The optimal cutoff value of JMJD2D expression was calculated based on the maximally selected rank statistics. Based on this analysis, the cohort of 479 patients was divided into high-JMJD2D (*n* = 207) and low-JMJD2D (*n* = 272) groups, with the optimal cutoff value set at 20.98 (Fig. 3C). Notably, high expression of JMJD2D was associated with poorer survival outcomes compared to low expression, as evidenced by a significant log-rank test result (*P* = 0.024) (Fig. 3D). Furthermore, correlation analyses between JMJD2D expression and clinical features revealed a significant association with the T stage (Fig. 3E). These findings underscore the potential prognostic significance of JMJD2D in LUAD and its potential role in tumor progression.

### Correlation of low JMJD2D expression with activation of Immune-Related pathways

**Fig. 4:**
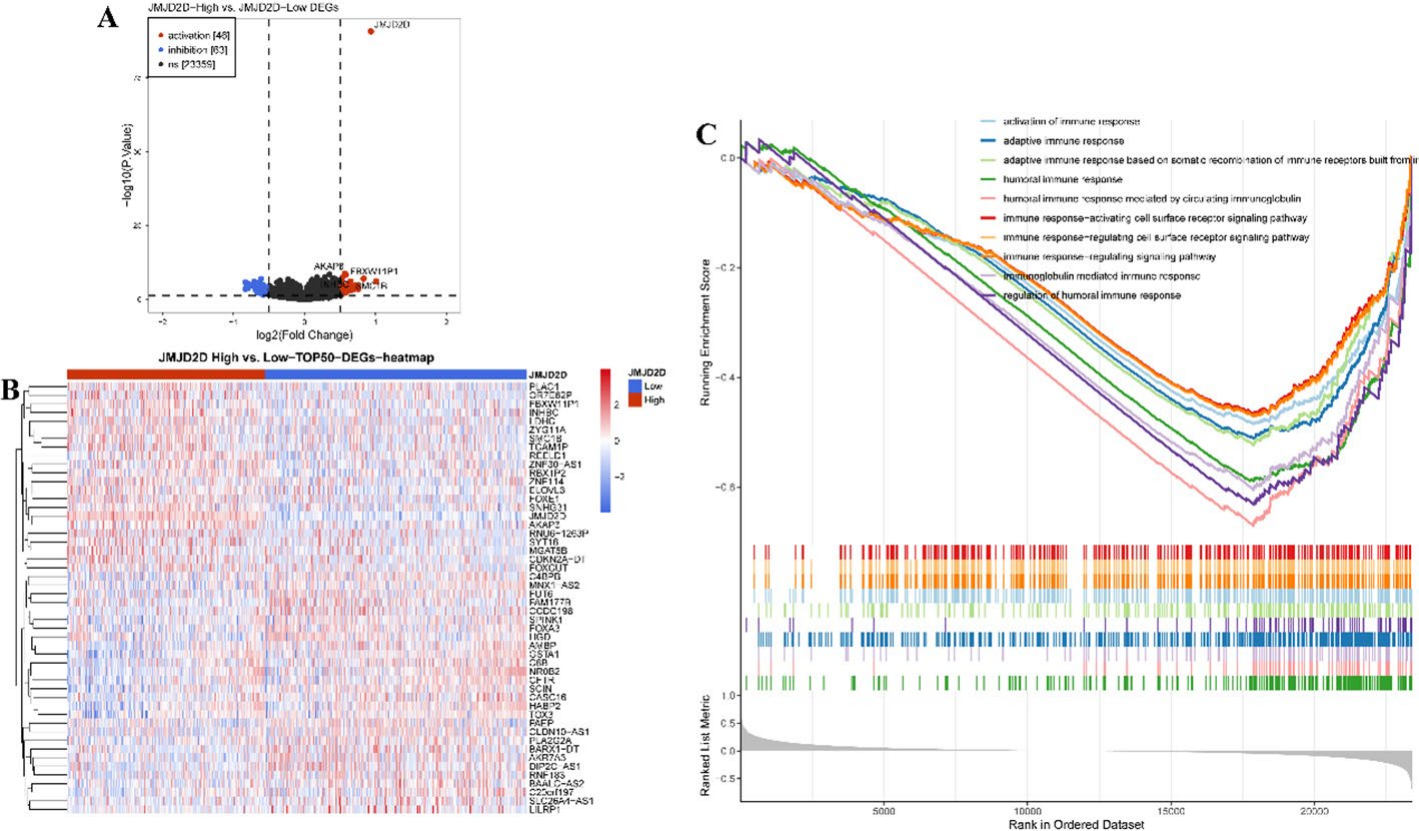
Correlation of low JMJD2D expression with activation of immune-related pathways. A. Volcano plots were generated to visualize the DEGs between the high-JMJD2D and low-JMJD2D groups, with the criteria of |log FC| > 0.5 and adjusted *P* < 0.05. B. A heatmap displaying the expression patterns of the top 50 DEGs between the high-JMJD2D and low-JMJD2D groups, ranked in the order of adjusted *P* -value. C. GSEA was conducted to identify enriched pathways, particularly focusing on immune-related pathways, in the high-JMJD2D and low-JMJD2D groups

Given the significant differences in prognosis associated with varying JMJD2D expression levels, we conducted differential gene expression analysis between the high-JMJD2D and low-JMJD2D groups. As shown in Fig. 4A, our analysis identified a total of 109 DEGs, comprising 46 upregulated and 63 downregulated DEGs, meeting the criteria of |log FC| > 0.5 and *P* < 0.05 (Table S1). Furthermore, the top 50 DEGs were ranked based on their P-values, as illustrated in Fig. 4B. Additionally, GSEA results revealed that the DEGs predominantly enriched in immune-related processes. Specifically, pathways associated with adaptive and humoral immune responses, positive regulation of immune responses, activation of the immune response, and immune response-regulating signaling pathways were significantly enriched in the low-JMJD2D group (Fig. 4C, Table S2). These findings suggest a potential link between JMJD2D expression levels and immune-related processes in LUAD, highlighting its potential role in tumor immunity.

### Correlation of JMJD2D expression with immune cell infiltration

**Fig. 5:**
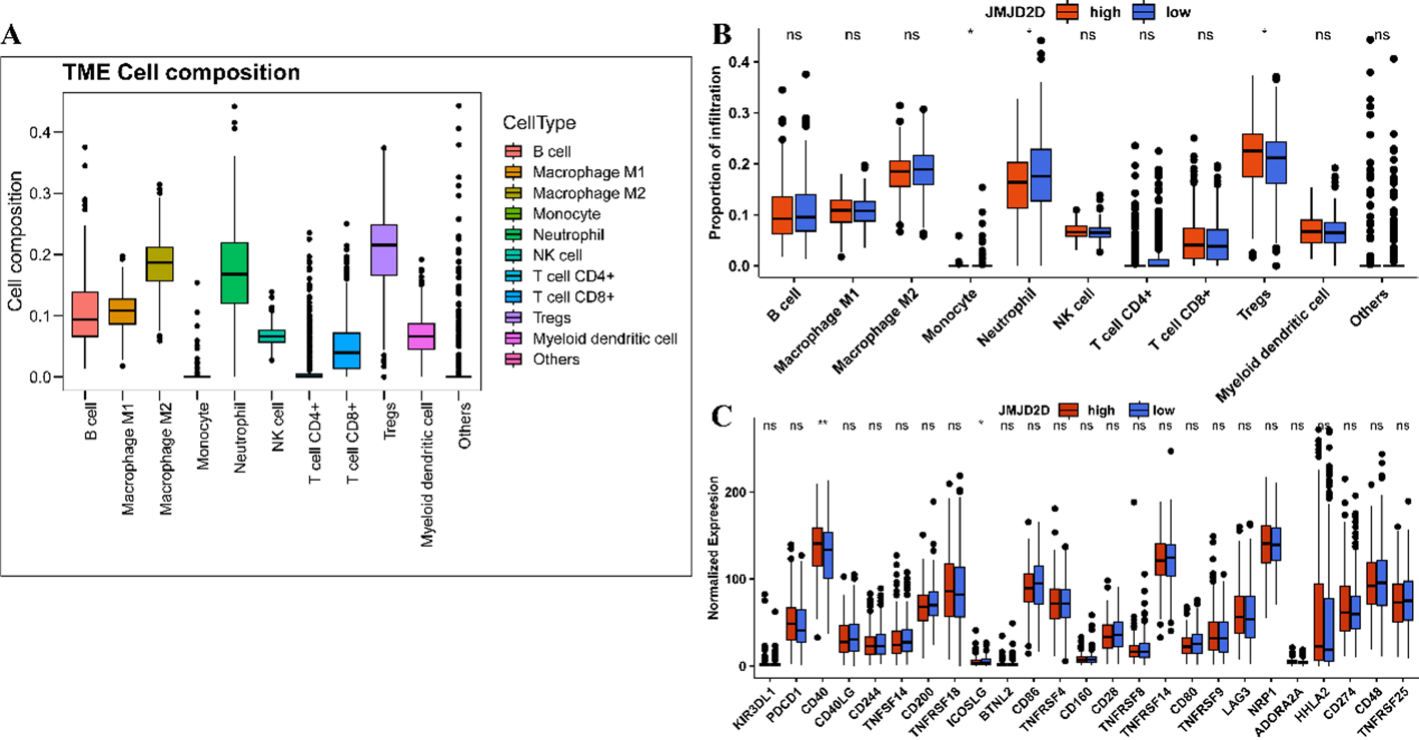
Correlation of JMJD2D expression with immune cell infiltration. A. Boxplots depicting the distribution of immune cell compositions within LUAD tumors. B. Boxplots illustrating the differences in the abundance of immune cells between the high-JMJD2D and low-JMJD2D groups. * *P* < 0.05. C. Boxplots demonstrating the variances in the expression levels of immune checkpoint-related genes between different JMJD2D expression groups. * *P* < 0.05, and ** *P* < 0.01

Given the significant association of JMJD2D with immune-related biological processes, we further investigated its relationship with tumor immune cell infiltration. As shown in Fig. 5A, various immune cell proportions, including B cells, macrophages (M1/M2), monocytes, neutrophils, NK cells, CD4 + and CD8 + T cells, regulatory T cells (Tregs), and myeloid dendritic cells, exhibited significantly higher levels compared to other cell types. Further analysis of immune cell distribution between the high-JMJD2D and low-JMJD2D groups revealed notable differences in monocytes, neutrophils, and Tregs (Fig. 5B). Specifically, Tregs were significantly increased, while monocytes and neutrophils were decreased in the high-JMJD2D group compared to the low-JMJD2D group. Moreover, we explored the expression of 25 Immune Checkpoint-Related Genes (ICRGs) between the high-JMJD2D and low-JMJD2D groups. The results unveiled significantly higher CD40 expression and lower ICOSLG expression in the high-JMJD2D group compared to the low-JMJD2D group (Fig. 5C). These findings provide further insight into the immune landscape of LUAD and underscore the potential role of JMJD2D in modulating immune cell infiltration.

### High JMJD2D expression correlates with high tumor mutation burden (TMB)

**Fig. 6:**
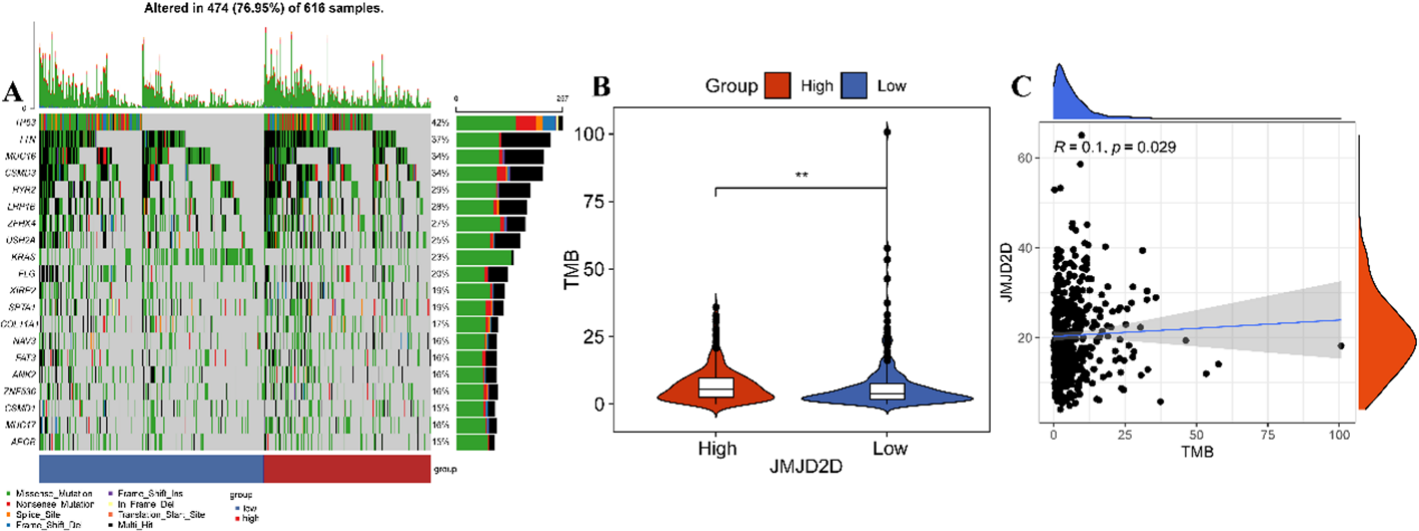
High JMJD2D expression correlates with high tumor mutation burden (TMB). A. Waterfall plot displaying the mutation status of tumor samples comparing high-JMJD2D and low-JMJD2D groups. B. Violin plots indicating the differences in TMB between high-JMJD2D and low-JMJD2D groups. ** *P* < 0.01. C. Scatter plots illustrating the correlation between JMJD2D expression levels and TMB

To investigate the relationship between JMJD2D expression and somatic mutations, we analyzed mutation data from 479 LUAD patients obtained from the TCGA database. As shown in Fig. 6A, the top 20 mutated genes between high-JMJD2D and low-JMJD2D groups, highlighting frequent genetic alterations. These genes included TP53 (42%), TTN (37%), MUC16 (34%), CSMD3 (34%), RYR2 (29%), LRP1B (28%), ZFHX4 (27%), USH2A (25%), KRAS (23%), FLG (20%), XIRP2 (19%), SPTA1 (19%), COL11A1 (17%), NAV3 (16%), FAT3 (16%), ANK2 (16%), ZNF536 (16%), CSMD1 (15%), MUC17 (16%), and APOB (15%). Moreover, our analysis revealed that TMB was significantly higher in the high-JMJD2D group compared to the low-JMJD2D group (Fig. 6B), Importantly, a weak positive correlation was observed between JMJD2D expression and TMB (Fig. 6C). These findings suggest that JMJD2D may be associated with increased genomic instability in LUAD, further underscoring its potential role in tumor progression.

### Correlation of JMJD2D with therapeutic responses

**Fig. 7:**
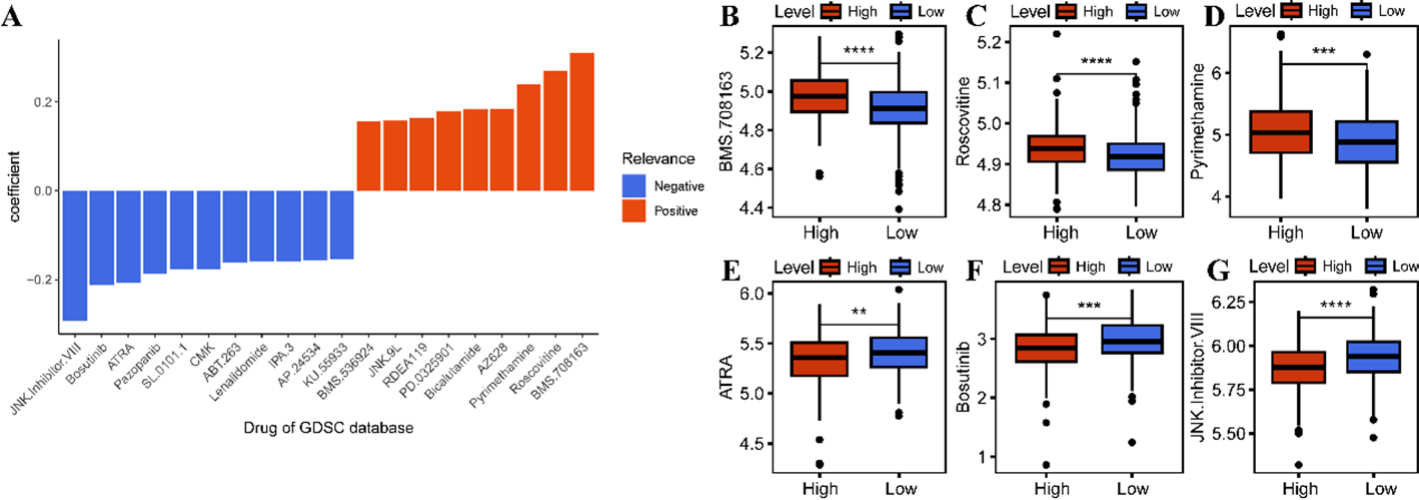
Correlation of JMJD2D with therapeutic responses. A. Correlation analysis depicting the relationship between JMJD2D expression and IC50 values of antitumor drugs sourced from the GDSC database. B-G. Boxplots showcasing the variances in IC50 values of antitumor drugs between high-JMJD2D and low-JMJD2D groups. * *P* < 0.05, ** *P* < 0.01, *** *P* < 0.001, and **** *P* < 0.0001

To further elucidate the relationship between JMJD2D expression and therapeutic responses in LUAD, we assessed drug sensitivity by calculating IC50 values for various chemotherapeutic agents using data from the GDSC database (Table S3). Correlation analysis between JMJD2D expression and IC50 values revealed distinct drug-specific associations. Specifically, JMJD2D expression was positively correlated with the sensitivity nine drugs (BMS.708163, Roscovitine, Pyrimethamine, AZ628, Bicalutamide, PD.0325901, RDEA119, JNK.9 L, BMS.536924), whereas a negative correlation was observed for eleven drugs (KU.55933, AP.24534, IPA.3, Lenalidomide, ABT.263, CMK, SL.0101.1, Pazopanib, ATRA, Bosutinib, JNK. Inhibitor. VIII) (Fig. 7A, Table S4). Further analysis focused on six drugs with a correlation coefficient exceeding 0.2, revealing significant differences in IC50 values between the high-JMJD2D and low-JMJD2D groups. Specifically, the high-JMJD2D group exhibited higher IC50 values for BMS.708,163, Roscovitine, and Pyrimethamine compared to the low-JMJD2D group (Fig. 7B-D). Conversely, the high-JMJD2D group displayed lower IC50 values for ATRA, Bosutinib, and JNK. Inhibitor. VIII compared to the low-JMJD2D group (Fig. 7E-G). These findings suggest that JMJD2D may play a crucial role in modulating drug response in LUAD, providing potential insights for precision therapy.

### Development of a JMJD2D-Related risk model for LUAD

**Fig. 8:**
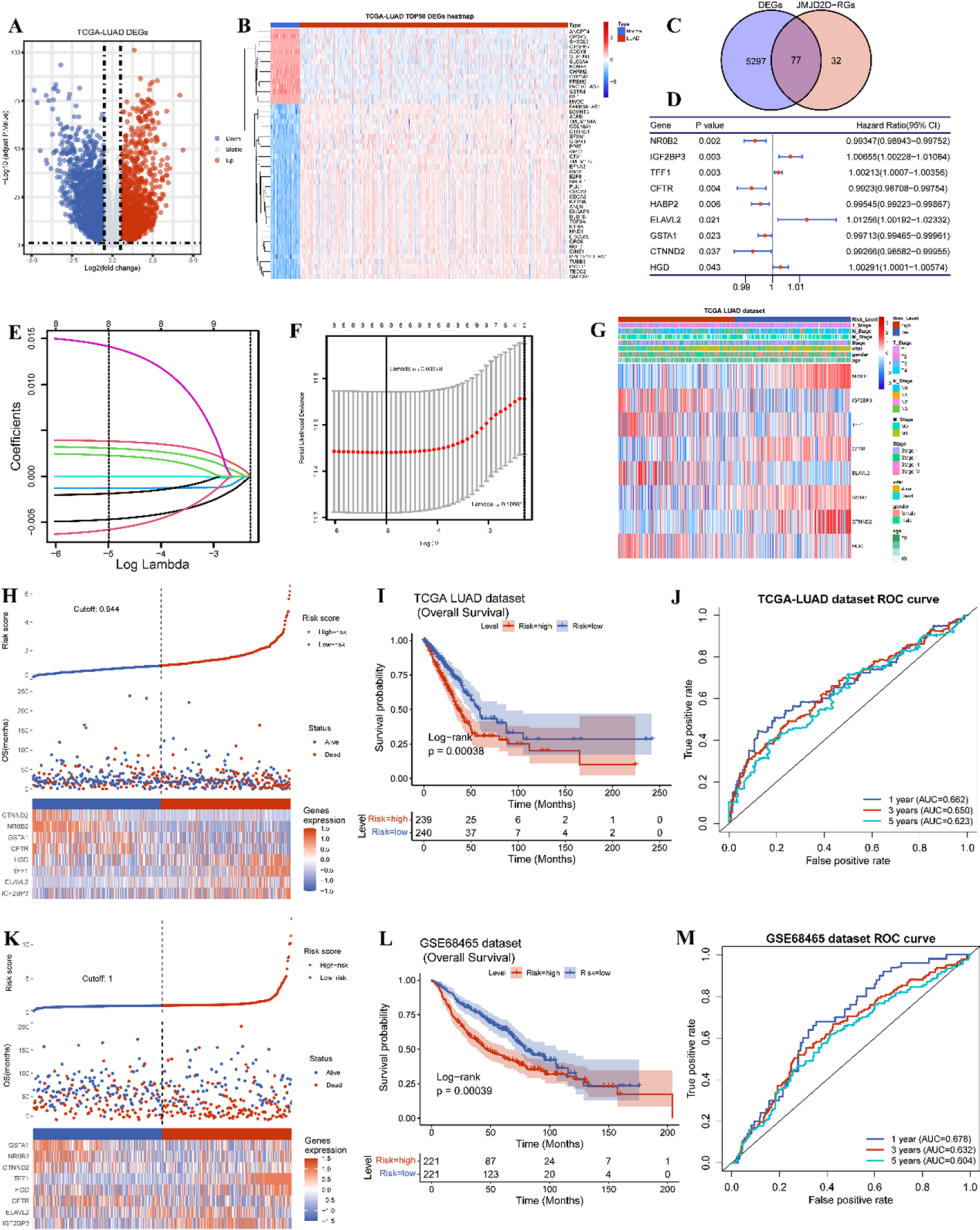
Development of a JMJD2D-related risk model for LUAD. A. Volcano plots illustrating the DEGs between LUAD tumors and normal samples, with the criterion of |log FC| > 0.5 and *P* < 0.05. B. Heatmap showcasing the top 50 DEGs between LUAD tumors and normal samples, ranked in the order of P-value. C. Venn plot displaying the intersection of genes obtained by overlapping DEGs in LUAD and JMJD2D-related DEGs (JMJD2D-RGs). D. Univariate Cox analysis of the prognostic genes in LUAD. E-F. LASSO regression analysis of the prognostic signature in LUAD. G. Heatmap visualizing the correlation of risk score with clinical characteristics (age, gender, pathologic stages, and AJCC stages). H and K. The distribution of risk score and survival status, as well as gene expression levels among patients in the TCGA-LUAD cohort and GSE68465 cohort, respectively. I and L. Kaplan-Meier survival plots of the patients with high-risk and low-risk in the TCGA-LUAD cohort and GSE68465 cohort, respectively. J and M. ROC curves of 1-, 3-, and 5-year survival prediction in the TCGA-LUAD cohort and GSE68465 cohort, respectively

Considering the multifaceted influences of JMJD2D on prognosis, tumor immune-related processes, TMB, and drug sensitivity in LUAD, we endeavored to develop a comprehensive JMJD2D-related prognostic signature. As shown in Fig. 8A-B, a total of 5374 DEGs (2951 upregulated and 2423 downregulated) between tumor and normal were identified with |log FC| > 0.5 and adjusted P-value < 0.05 (Table S5). Subsequently, we overlapped these DEGs with the 109 JMJD2D-related DEGs, resulting in the selection of 77 DE-JMJD2D-RGs for constructing the prognostic signature (Fig. 8C). Utilizing Kaplan-Meier survival analysis, we screened nine prognostic DE-JMJD2D-RGs with a P-value < 0.05 (Fig. 8D). Next, using LASSO regression analysis, we further refined the prognostic genes, ultimately identifying eight genes (NR0B2, IGF2BP3, TFF1, CFTR, ELAVL2, GSTA1, CTNND2, HGD) for inclusion in the prognostic signature (Fig. 8E-F). The risk score was calculated using the following formula: RiskScore = NR0B2*-0.005066 + IGF2BP3*0.0039992 + TFF1*0.0033018 + CFTR*(-0.0013022) + ELAVL2*0.015465 + GSTA1*(-0.0021139) + CTNND2*(-0.0065844) + HGD*0.002528 (Table 1). Subsequently, 479 patients from the TCGA-LUAD cohort were stratified into high- and low-risk groups using the median risk score of 0.944 (Fig. 8H). Kaplan-Meier survival analysis revealed poorer survival outcomes in the high-risk group compared to the low-risk group (Fig. 8I). Additionally, the predictive accuracy of the prognostic signature was evaluated using ROC curves for 1-, 3-, and 5-year OS, yielding AUC values of 0.662, 0.650, and 0.623, respectively (Fig. 8J). Furthermore, external validation was performed using the GSE68465 dataset, where 442 patients were divided into high- and low-risk groups based on the median risk score of 1.25 (Fig. 8K). Consistent with the TCGA-LUAD cohort, the high-risk group exhibited worse survival outcomes than the low-risk group (Fig. 8L). The AUC values of the ROC curves for 1-, 3-, and 5-year OS were 0.678, 0.632, and 0.604, respectively (Fig. 8M). Additionally, we investigated the correlation between the risk score and clinical features, revealing differences in gender, survival status, overall survival time, pathologic stages, and AJCC stages between the high-risk and low-risk groups (Fig. 8G; Table 2). Overall, the JMJD2D-related prognostic signature demonstrates promising predictive performance and clinical utility in prognostic assessment for LUAD patients.

**Table 1:** LASSO regression analysis selects the prognostic genes in the TCGA-LUAD cohort

ID	Coef	Exp (coef)	Se (coef)	Z
NR0B2	-0.005066	0.9949468	0.0021856	-2.318
IGF2BP3	0.0039992	1.0040072	0.0024611	1.625
TFF1	0.0033018	1.0033073	0.0008914	3.704
CFTR	-0.0013022	0.9986987	0.0030442	-0.428
ELAVL2	0.015465	1.0155852	0.0059182	2.613
GSTA1	-0.0021139	0.9978883	0.0015176	-1.393
CTNND2	-0.0065844	0.9934373	0.0035504	-1.855
HGD	0.002528	1.0025312	0.0016827	1.502

**Table 2:** Correlation of the risk score and clinical features in the TCGA-LUAD cohort

Variables	Total	Risk score		*P*-value
		High	Low	
Age (year)				
Mean (SD)	65.3 (± 10.1)	64.9 (± 10.5)	65.7 (± 9.6)	0.48
Gender				
Female	260 (54.3%)	117 (49.0%)	143 (59.6%)	0.022
Male	219 (45.7%)	122 (51.0%)	97 (40.4%)	
Survival status				
Alive	302 (63.0%)	134 (56.1%)	168 (70.0%)	0.002
Dead	177 (37.0%)	105 (43.9%)	72 (30.0%)	
OS (Months)				
Mean (SD)	876.9 (± 874.6)	807.9 (± 805.9)	945.6 (± 934.7)	0.032
Pathologic stage				
Stage I	259 (54.1%)	112 (46.9%)	147 (61.3%)	0.015
Stage II	117 (24.4%)	67 (28.0%)	50 (20.8%)	
Stage III	78 (16.3%)	44 (18.4%)	34 (14.2%)	
Stage IV	25 (5.2%)	16 (6.7%)	9 (3.8%)	
Pathologic T				
T1	164 (34.5%)	61 (25.7%)	103 (43.1%)	< 0.001
T2	251 (52.7%)	136 (57.4%)	115 (48.1%)	
T3	44 (9.2%)	30 (12.7%)	14 (5.9%)	
T4	17 (3.6%)	10 (4.2%)	7 (2.9%)	
Pathologic N				
N0	311 (66.2%)	146 (62.1%)	165 (70.2%)	0.16
N1	90 (19.1%)	51 (21.7%)	39 (16.6%)	
N2	67 (14.3%)	36 (15.3%)	31 (13.2%)	
N3	2 (0.4%)	2 (0.9%)	0 (0.0%)	
Pathologic M				
M0	316 (92.9%)	157 (91.3%)	159 (94.6%)	0.29
M1	24 (7.1%)	15 (8.7%)	9 (5.4%)	

### Construction of a predictive nomogram

**Fig. 9:**
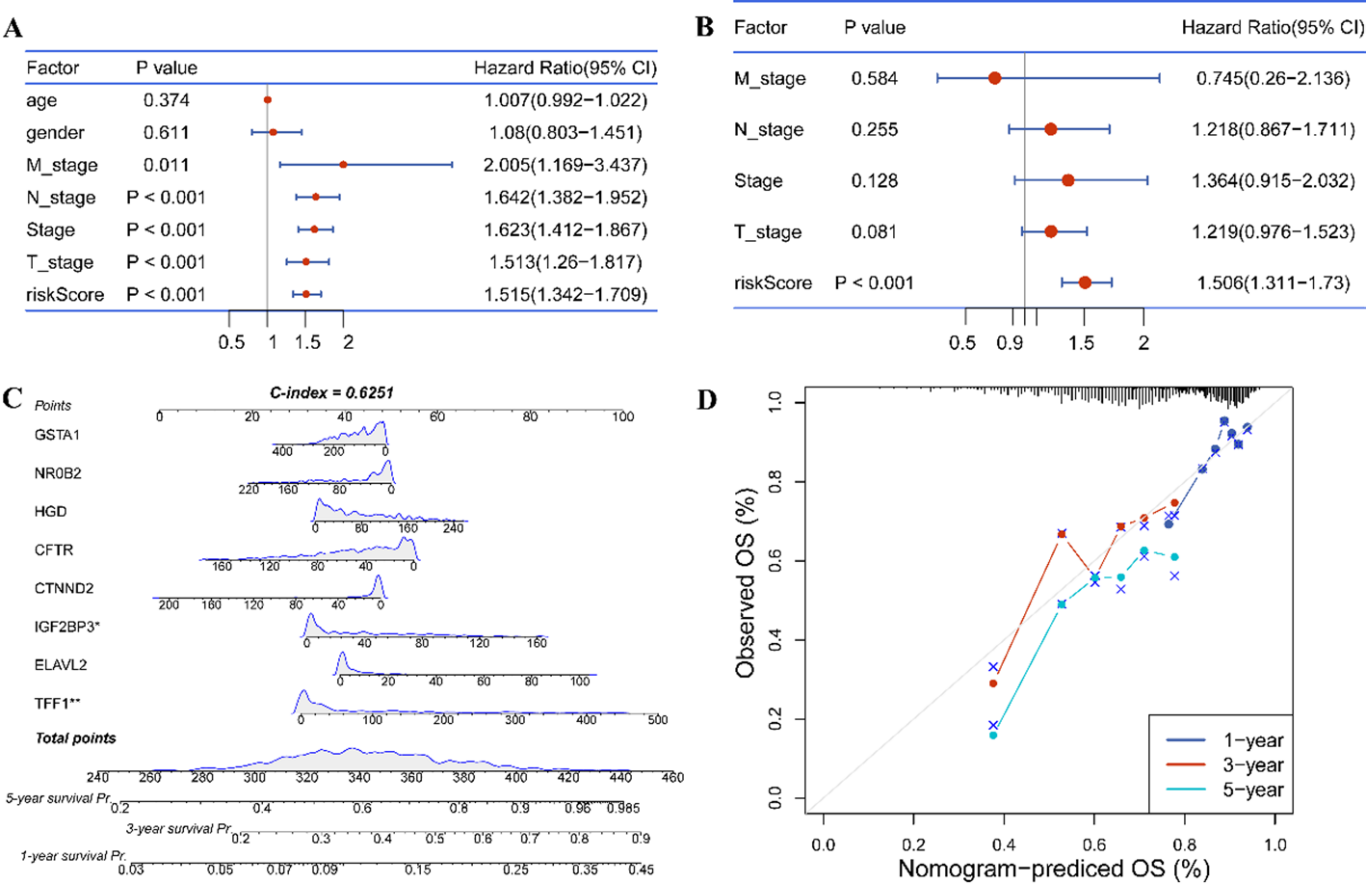
Construction of a predictive nomogram. A-B. Results of Univariate and Multivariate Cox analyses depicting the association of the risk score and clinical characteristics with Overall Survival (OS). C. A predictive nomogram designed to forecast possible 1-, 3-, and 5-year survival time. D. Calibration curves illustrating the survival probability at 1-, 3-, and 5-years, verifying the accuracy of the predictive nomogram

The risk score, age, gender, pathologic stages, and AJCC stages, were subjected to univariate and multivariate Cox regression analyses to identify the independent factors for LUAD. The results indicated that risk score was only independent factor for prognosis in LUAD (Fig. 9A-B). Subsequently, a nomogram was constructed by integrating the risk score and relevant risk factors (Fig. 9C). The C-index was determined to be 0.6251, indicating moderate discriminative ability. Additionally, calibration curves for survival probability at 1-, 3-, and 5-years demonstrated the accurate predictive performance of the nomogram across both short- and long-term intervals (Fig. 9D). Overall, these findings underscore the utility of the risk score as an independent prognostic factor for LUAD, and the nomogram provides a valuable tool for individualized prognostic assessment in clinical practice.

### Correlation of risk score with immune characteristics

**Fig. 10:**
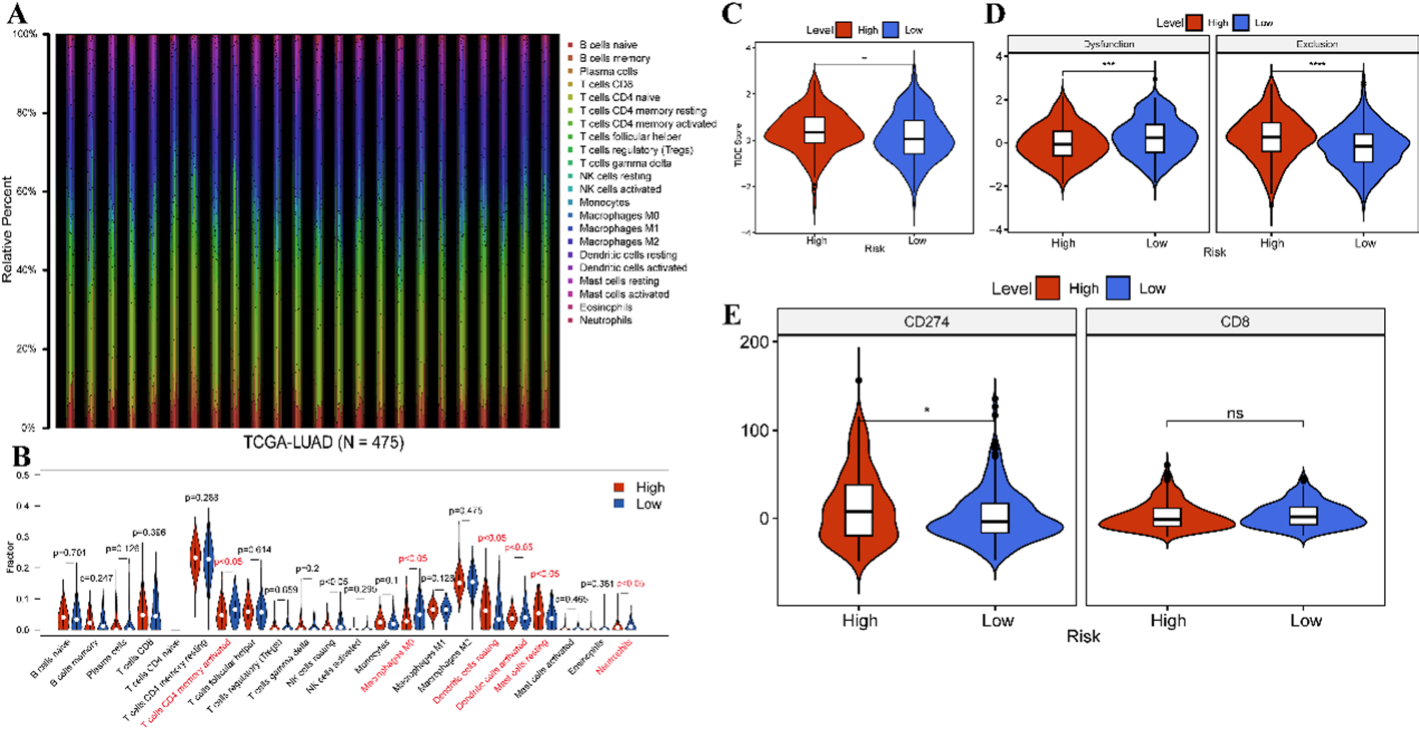
Correlation of risk score with immune characteristics. A. Heatmap depicting the enrichment of infiltrating immune cells in LUAD patients. B. Violin plots showcasing the differences in TIDE score between high-risk and low-risk groups in the TCGA-LUAD cohort. C-D. Violin plots demonstrating the disparities in dysfunction and exclusion between high-risk and low-risk groups in the TCGA-LUAD cohort. ** *P* < 0.01, *** *P* < 0.001, and **** *P* < 0.0001. E. Violin plots illustrating the differential expression of CD274 and CD8 between high-risk and low-risk groups in the TCGA-LUAD cohort. * *P* < 0.05

Given the association of the risk score with poor clinical outcomes of LUAD patients, we investigated risk score correlation with immune characteristics. Initially, we utilized the CIBERSORT algorithm to determine the distribution of 22 types of immune cells in tumor samples (Fig. 10A). Subsequently, we compared the immune cell distribution between high- and low-risk groups, revealing a significant decrease in the fraction of activated CD4 memory T cells, Macrophages M0, and activated dendritic cells (DCs), alongside increased resting DCs, resting mast cells, and neutrophils in the high-risk group compared to the low-risk group (Fig. 10B). Moreover, we observed higher TIDE score and exclusion score, but lower dysfunction scores in the high-risk group relative to low-risk group (Fig. 10C-D). Besides, higher expression of CD274 was detected in the high-risk group compared to the low-risk group (Fig. 10E). These findings suggest that high-risk scores may be linked to immune suppression in LUAD, potentially contributing to a less favorable prognosis.

## Discussion

With the development of next-generation sequencing (NGS), numerous crucial molecular biomarkers have been identified in the diagnosis and treatment of NSCLC, including alterations in EGFR, BRAF, MET, ALK, ROS1, RET, and NTRK, along with the corresponding kinase inhibitors [8, 29, 45]. Despite significant progress has been made in the diagnosis and treatment of NSCLC over recent decades, the overall survival rates for patients with NSCLC remain suboptimal. Consequently, the search for more sensitive biomarkers to improve the diagnosis and treatment of NSCLC continues to be a critical challenge in enhancing patient survival outcomes.

In recent years, accumulating evidence has highlighted the pivotal role of epigenetic modifications in cancer initiation and progression [46]. Reversible histone methylation, is a crucial process involved in tumor onset and advancement [47–49]. Numerous studies have elucidated the significant involvement of JMJD2D in regulating tumor initiation, progression, and drug resistance, positioning it as a promising therapeutic target across various cancers [50–52]. In our present study, we observed significant overexpression of JMJD2D in BLCA, COAD, HNSC, LUAD, LUSC, and STAD. Elevated JMJD2D expression was closely associated with poorer survival outcomes in LUAD patients, inhibition of immune-related pathways, heightened TMB, and resistance to certain anti-tumor drugs. Previous research has indicated that JMJD2D facilitates CRC immune evasion by inducing PD-L1 overexpression [24]. Here, we not only found the high-JMJD2D-induced inhibition of immune-related pathways but also noted an upregulation of CD40 expression, suggesting that JMJD2D may contribute to immune escape in LUAD, thereby accelerating tumor progression. Additionally, JMJD2D has been implicated in DNA damage response (DDR), linking it to cancer-relevant epigenetic regulation and genome stability [53–56]. Similarly, we also found some mutated genes associated with JMJD2D and observed a weak correlation between high JMJD2D expression and elevated TMB. Furthermore, we found that JMJD2D conferred resistance to BMS.7.8163, Roscovitine, and Pyrimethamine, while displaying sensitivity to ATRA, Bosutinib, and JNK. Inhibitor. VIII. These findings collectively suggest that JMJD2D may serve as a potential biomarker for guiding anti-tumor drug selection.

Based on the connection between JMJD2D and tumorigenesis, particularly in LUAD progression, we developed a risk score model using JMJD2D-related genes, including NR0B2, IGF2BP3, TFF1, CFTR, ELAVL2, GSTA1, CTNND2, and HGD. Among these genes, NR0B2, CFTR, GSTA1, and CTNND2 were found to exert a protective role, whereas IGF2BP3, TFF1, ELAVL2, and HGD were associated with higher-risk scores. NR0B2 has been identified as a favorite prognosis factor in liver cancer [57]. CFTR, a cAMP-activated chloride channel, regulates fluid homeostasis and transports relevant substrates [58]. Alterations in CFTR are implicated in various tumors, with dysregulation linked to more aggressive lung cancers [59, 60]. GSTA1, a component of glutathione S-transferases (GSTs), participates in carcinogen detoxification and plays a crucial role in carcinogenesis [61]. Clinical trials have shown that genetic polymorphisms in GSTA1 are linked to lung cancer occurrence [62]. CTNND2 is overexpressed in lung cancer, promoting tumorigenesis and metastasis [63, 64].

In contrast, IGF2BP3, an N6-Methyladenosine (m6A) reader, acts as an oncogene gene in lung cancer [65, 66]. TFF1 overexpression has been observed in various cancers, including breast, colonic, pancreatic, ovary, prostate, thyroid, and lung cancers, is associated with chemoresistance, metastasis, and poor prognosis [67–70]. ELAVL2, although its role in lung cancer is less explored, has been reported as an oncogene promotes ovarian cancer cells resistant to paclitaxel [71]. HGD, another relatively unexplored gene in cancers, is involved in cholangiocarcinoma genesis [72], and has been identified as a candidate co-expressed with B-type Raf kinase (BRAF) V600E mutation in papillary thyroid carcinoma [73]. Collectively, these eight genes hold promise as potential biomarkers for molecular diagnosis and treatment strategies in LUAD. Further research on their roles and mechanisms in LUAD pathogenesis may provide valuable insights into the disease’s management.

Based on the risk score, we also explored the association of immune characteristics with the risk score in LUAD. The findings revealed a decrease in the proportions of activated memory CD4 + T cells, Macrophages M0, and activated DCs, along with an increase in resting DCs, resting mast cells, and neutrophils in samples with a high-risk score. Moreover, higher TIDE score and exclusion score, as well as overexpression of CD274, were associated with a high-risk score, suggesting potential involvement of high-risk scores in immunosuppression.

Despite the comprehensive exploration of the role and regulatory mechanisms of JMJD2D in LUAD in our study, there are still some limitations that need to be addressed. First, a larger sample cohort is needed to further validate our findings and enhance the statistical power of the analysis. Second, experimental studies are required to confirm the regulatory mechanisms proposed in this study, providing deeper insights into the functional roles of JMJD2D in LUAD.

## Conclusion

In summary, our study elucidated the oncogenic role of JMJD2D in LUAD, highlighting its involvement in tumorigenesis, progression, antitumor resistance, and poor prognosis. Additionally, we identified a JMJD2D-related prognostic signature and developed both a risk model and predictive model for LUAD, offering potential tools for personalized prognosis and therapeutic strategies in LUAD patients.

## Supplementary Information

Supplementary Material 1.

## Data Availability

Publicly available datasets were analyzed in this study. This data can be found here, TCGA (https://portal.gdc.cancer.gov/), UCSC Xena (https://xena.ucsc.edu/), GEO (https://www.ncbi.nlm.nih.gov/geo/).
